# Patterns in Place of Cancer Death in the State of Qatar: A Population-Based Study

**DOI:** 10.1371/journal.pone.0109615

**Published:** 2014-12-23

**Authors:** Hassan Mohsen, Pascale Haddad, Ayman Allam, Azza Hassan

**Affiliations:** 1 Weill Cornell Medical College in Qatar, Doha, Qatar; 2 National Center for Cancer Care and Research, Doha, Qatar; 3 Cancer Management & Research, Medical Research Institute, Alexandria University, Egypt; West Virginia University, United States of America

## Abstract

**Background:**

International studies show that most people prefer to die at home; however, hospitals remain the most common place of death (PoD). This study aims to investigate the patterns in PoD and the associated factors, which are crucial for end-of-life cancer care enhancement.

**Method:**

This retrospective, population-based study analyzed all registered cancer deaths in Qatar between January 1, 2006 and December 31, 2012 (n = 1,224). The main outcome measures were patient characteristics: age, gender, nationality, cancer diagnosis, year of death, and PoD. Time trends for age-standardized proportions of death in individual PoDs were evaluated using chi-square analysis. Odds ratio (OR) were determined for variables associated with the most preferred (acute palliative care unit [APCU] and hematology/oncology ward) versus least preferred (ICU and general medicine ward) PoDs in Qatar, stratified by nationality.

**Results:**

The hematology/oncology ward was the most common PoD (32.4%; 95% CI 26.7–35.3%) followed by ICU (31.4%; 95% CI 28.7–34.3%), APCU (26.9%; 95% CI 24.3–29.6%), and general medicine ward (9.2%; 95% CI 7.6–11.1%). APCU trended upward (+0.057/year; p<0.001), while the hematology/oncology ward trended downward (−0.055/year; p<0.001). No statistically significant changes occurred in the other PoDs; home deaths remained low (0.4%; 95% Cl 0.38–0.42). Qataris who died from liver cancer (OR 0.23) and aged 65 or older (OR 0.64) were less likely to die in the APCU or hematology/oncology ward (p<0.05). Non-Qataris who died from pancreatic cancer (OR 3.12) and female (OR 2.05) were more likely to die in the APCU or hematology/oncology ward (p<0.05). Both Qataris and non-Qataris who died from hematologic malignancy (OR 0.18 and 0.41, respectively) were more likely to die in the ICU or general medicine ward (p<0.05).

**Conclusion:**

A high percentage of cancer deaths in Qatar occur in hospital. As home was the preferred PoD for most people, effective home care and hospice programs are needed to improve end-of-life cancer care.

## Introduction

Cancer is a leading cause of death worldwide, and the number of deaths from cancer is expected to increase in the coming years. In 2012, nearly 8 million people died of cancer worldwide and by 2030 the World Health Organization (WHO) estimates that 21 million people will develop cancer and 13 million will die from cancer worldwide, translating to an increase of approximately 60% in cancer deaths [1]. In spite of extensive research into the management and prevention of cancer, the 5-year survival rate for all cancers is only 50–70% in developed countries [2]–[3]. Although improvements in early diagnosis and management have resulted in a high cure rate for some cancer types (e.g., breast, lymphoma, and prostate), there are other cancer types (e.g., pancreas, lung, liver, and esophagus) for which the survival rate remains low [2]–[3]. Moreover, the number of deaths from cancer is projected to increase worldwide, with the aging population and increase in chronic disease prevalence [4]. As a result, countries with aging populations are inevitably challenged with an increasing need for end-of-life care [5]. This has prompted the WHO to spearhead international recommendations on end-of-life care planning, which heavily emphasize patient preference for the place of care and death at the end of life [6]–[8].

Major studies on patient preferences from several countries have revealed that most people prefer to die at home with the provision of adequate care [9]–[11]. For most people, home represents a place of connection and comfort, a sense of normalcy and familiarity, and the opportunity to be around loved ones while enjoying a “normal” life [12]–[14]. As a result, several countries have made considerable efforts to enhance home care [15]. Despite these efforts, however, studies have shown that most cancer patients in Europe, North America, Taiwan, and Australia die in hospitals [16]–[22].

Meeting people's preferences for end-of-life care has considerable economic implications for healthcare systems [23]–[24]. Approximately one-third of the annual net cancer care costs are spent during the final year of a patient's life, with projections showing increasing annual expenditure as costs of cancer management rise and more people reach an older age [25]–[26]. Other research has shown that end-of-life care by hospital in-patient services is associated with costs that are three times higher than those of community palliative care services [27]. Importantly, higher spending and more aggressive treatments during the last year of life do not yield better outcomes for patients [28]–[32]. In fact, cancer patients who die in the hospital or ICU have a worse quality of life, and their grieving caregivers are at an increased risk of post-traumatic stress and prolonged grief disorders compared to patients who die at home with augmented hospice services [33]–[35]. Therefore, minimizing unnecessary hospital deaths and optimizing home and hospice utilization, in accordance with patient preferences, has become a crucial issue for healthcare policy initiatives in many countries [36]–[41].

Qatar is a peninsula covering an area of 11,571 km^2^, with Saudi Arabia as its sole land border to the south and the Persian Gulf surrounding the remaining territory. Qatar has the world's third largest natural gas reserves, which has empowered it to become the world's richest country per capita [42]. Healthcare is provided free of charge to its population of 2,045,239 individuals [43].

The National Center for Cancer Care and Research (NCCCR) is the only tertiary cancer care center in Qatar and was established in 2004 under the Hamad Medical Corporation (HMC), which manages a total of 8 highly specialized hospitals. The NCCCR currently comprises a 58-bed hematology/oncology ward for patients undergoing active cancer management. In July 2008, a 10-bed specialized acute palliative care unit (APCU) was established to serve as an acute unit where patients are admitted for acute symptom management. At this time, no other alternative palliative care models are offered, including home care, hospice, or subacute or chronic palliative care services. Cancer is the third leading cause of death in Qatar, accounting for almost 10% of all deaths [44].

In order to improve the quality of end-of-life cancer care and enable more people to die in their preferred place, it is crucial to analyze the changing patterns and factors associated with PoD of cancer patients. To the best of our knowledge, this is the first population-based study in the Middle East analyzing the PoD of cancer patients over a several-year period.

This study sought to investigate the changes over time in the following: (i) places of cancer death, (ii) patient profile and cancer diagnosis, and (iii) factors associated with place of cancer death and their relative importance. Then to (iv) compare our results with findings in other countries.

## Methods

### Data Sources

All deaths between 2006 and 2012 where cancer was the underlying cause of death were extracted from the National Registry of Deaths Database. By law in Qatar, a death must be registered, and the underlying cause of death recorded to approve burial of the deceased. The underlying cause of death is recorded in the database using the 10^th^ edition of the International Classification of Diseases (ICD-10) codes.

### Study Design and Study Population/Cohort Selection

This was a retrospective, population-based study of a decedent cohort of all patients who died as a result of any cancer type in the State of Qatar between January 1, 2006 and December 31, 2012. In the State of Qatar and other Gulf Cooperation Council (GCC) countries, the pediatric age group is defined as up to 14 years of age; therefore, we limited the analysis to individuals who died 15 years of age or older.

### Variables

The place of death (PoD) was grouped into six categories: acute palliative care unit (APCU), hematology/oncology ward, general medicine (inpatient) ward, ICU, abroad, and other (home, long-term care facility, private hospital, or ER). We grouped the other location categories together because the number of deaths occurring at those locations was very small. Explanatory variables included the following: age at death (15–24, 25–54, 55–64, 65–74, 75–84, and 85+ years), sex (male and female), cancer type (See Table 1 for ICD 10 codes), year of death, and nationality. We analyzed age as an ordered six-category variable rather than as a continuous variable to facilitate interpretation and comparison with other studies; the cut-off boundaries were chosen based on the data distribution.

**Table 1:** Demographic characteristics of all deaths with cancer as the underlying cause of death in Qatar, 2006–2012 (data are shown as percentages, except for the total # of deaths/year).

		Year of Death							
		2006	2007	2008	2009	2010	2011	2012	Total
**All**	Total # of deaths	129	125	164	195	197	189	225	1224
**Sex**	Male	58.9	60.0	53.7	56.9	50.8	55.0	53.8	55.1
	Female	41.1	40.0	46.3	43.1	49.2	45.0	46.2	44.9
**Age**	15–24 years	2.3	4.8	2.4	2.6	2.0	3.2	1.8	2.6
	25–34 years	3.9	3.2	6.1	6.2	5.6	5.3	3.1	4.8
	35–44 years	10.9	7.2	7.3	14.9	11.2	9.0	8.4	10.0
	45–54 years	20.9	21.6	15.2	20.0	20.3	19.6	23.1	20.2
	55–64 years	20.9	30.4	31.1	18.5	28.4	27.0	26.2	26.0
	65–74 years	27.1	16.0	21.3	24.6	19.3	19.0	20.9	21.2
	75–84 years	10.9	13.6	14.6	10.8	12.2	13.8	13.3	12.7
	> 85 years	3.1	3.2	1.8	2.6	1.0	3.2	3.1	2.5
**Nationality**	Qatari	36.4	55.2	47.6	40.0	45.7	45.0	45.3	44.9
	Non-Qatari	63.6	44.8	52.4	60.0	54.3	55.0	54.7	55.1
**Cancer Diagnosis**	Bladder	1.6	1.6	3.7	2.1	3.0	1.6	0.9	2.0
	Brain	7.0	2.4	1.8	7.2	4.1	5.3	2.2	4.2
	Breast	12.4	11.2	10.4	14.4	13.7	13.2	17.3	13.6
	Ca unknown*	0.8	3.2	1.8	1.0	2.5	1.6	4.9	2.4
	Cervix	1.6	2.4	1.2	1.0	1.5	1.1	0.9	1.3
	Colorectal	6.2	8.0	9.8	3.6	10.7	12.7	8.0	8.5
	Esophagus	4.7	1.6	0	0.5	2.0	3.2	0.9	1.7
	Gallbladder	2.3	1.6	1.8	1.0	3.0	1.1	1.8	1.8
	Head & Neck	3.9	6.4	4.9	3.6	1.5	3.2	5.3	4.0
	Hematologic	16.3	16.0	14	17.4	14.2	12.2	11.1	14.2
	Kidney	4.7	2.4	1.8	2.6	1.5	3.2	0.9	2.3
	Liver	7.8	8.8	9.1	5.6	6.6	10.6	6.7	7.8
	Lung	13.2	12.8	16.5	13.3	16.8	10.6	10.2	13.2
	Melanoma	0.8	0	0	1.5	0.5	0.5	0.4	0.6
	Mesothelioma	0	0	0	0	0.5	0.5	0.4	0.2
	Neuroendocrine	0.8	0	0	0.5	0	0	0.9	0.3
	Omentum	0	0.8	0	0	0	0.5	0	0.2
	Ovary	2.3	4.0	1.8	5.1	3	1.1	3.6	3.0
	Pancreas	3.1	4.8	6.1	6.2	4.1	5.8	6.7	5.4
	Prostate	0.8	1.6	4.9	4.1	1.5	2.6	4.0	2.9
	Sarcoma	1.6	3.2	2.4	3.1	1.5	2.1	1.3	2.1
	SCC Skin‡	0	0	0	0	0.5	0	0.4	0.2
	Small Intestine	0	0	0	1.0	1.0	0	0.4	0.4
	Stomach	7.0	4.8	4.3	4.6	4.6	4.8	8.0	5.5
	Testis	0.8	0	0	0	0	0.5	0	0.2
	Thymoma	0	0	0	0	0	0.5	0	0.1
	Thyroid	0	0.8	1.2	0	1.0	0.5	0.4	0.6
	Uterus	0.8	1.6	1.8	0	0.5	1.1	2.2	1.1
	Vagina	0	0	0.6	0.5	0	0	0	0.2
									
**PoD**	Hematology/oncology ward	55.8	64.0	36.6	22.1	21.3	7.4	17.8	28.7
	Acute palliative care unit	0	0	7.3	25.1	31.5	43.4	38.2	23.8
	General medicine ward	13.2	6.4	13.4	6.2	3.0	8.5	8.4	8.2
	Intensive care unit	25.6	18.4	29.9	35.9	27.9	27.5	25.8	27.8
	Abroad†	1.6	5.6	6.7	6.7	8.1	6.9	4.9	6.0
	Other§	3.9	5.6	6.1	4.1	8.1	6.3	4.9	5.6

* Ca unknown: cancer of unknown orgin.

‡ SCC Skin: squamous cell carcinoma of the skin.

† Abroad refers to individuals who died abroad and brought home (Qatar) to their family for burial.

§ Places with low percentages were grouped together into one group (Other). This includes: other government facilities belonging to Hamad Medical Corporation, private hospitals and patients who died at home or in the emergency room.

### Statistical Analysis

Descriptive statistics were calculated as percentages for total deaths, sex, nationality and cancer diagnosis by year. We then calculated the age-adjusted proportion of deaths according to the PoD. The proportions were standardized using the 2005–2010 mortality structure for Western Asia from the United Nations standard population [45] and then plotted against the year of death. We then conducted a chi-squared trend analysis to test for changes in the rate of death per PoD throughout the whole period of the study (2006-2012). The rate of change per PoD along with its standard error, 95% confidence interval and p-value were evaluated.

To investigate the different factors associated with the PoD, bivariate and multivariable logistic regression were used. The dependent variable was the PoD, which was binary with two categories: APCU and hematology/oncology ward (1) versus general medicine ward and ICU (0). Given the lack of alternative palliative care modules for providing patients with the required palliative care, families tend to believe that the hospital setting, where trained physicians can provide adequate and specialized care, is the ideal place for their relatives in the end-of-life period [53]. Therefore, APCU and hematology/oncology ward (together they makeup the NCCCR) were considered to be the most preferred PoD, whereas general medicine ward and ICU (both located at HGH, which is the main branch of Hamad Medical Corporation) to be the least preferred PoD. We assessed the association of each of the independent variables with the PoD using a bivariate analysis and report the odds ratios (OR) along with the 95%CI and p-value. The independent variables were the following: age (i.e., less than 65 years and at least 65 years), sex (i.e., male and female), nationality (i.e., Qatari and non-Qatari), cancer diagnosis (i.e., colorectal, hematological, breast, lung, liver, stomach, pancreas and other) and year of death (before 2009 and after 2009). We chose to study before and after 2009 because this year marks the point when the APCU was established. We then constructed a model including all of the independent variables in a multivariate logistic regression. Adjusted ORs along with the 95% CI and p-values were analyzed.

We then tested for interaction terms between nationality and sex in addition to nationality and age, separately. The interaction terms were found to be significant and the likelihood ratio test, comparing the original model (with no interaction terms) with the model with 2 interaction terms (nationality and sex, and nationality and age), was also found to be significant. Rather than reporting the model with interaction terms, we stratified our model by nationality. We used adjusted ORs, which were stratified by nationality along with the 95% CI and p-values. p≤0.05 was considered significant. All analyses were conducted using Stata version 13.

### Ethics and Permission

This study was approved by The Research Ethics Committee, HMC Medical Research Center, Qatar.

## Results

### Descriptive Analysis

In total, 1,224 cancer deaths occurred during the period of January 1, 2006 to December 31, 2012 (Table 1). The annual number of cancer deaths progressively increased from 129 in 2006 to 225 in 2012. The mean age was 58.60 years with an SD of 15.37. More men than women died of cancer over the entire study period, with males constituting 55.07% of deaths and females 44.93%, in addition to 44.85% being Qatari and 55.15% being non-Qatari. The five most common causes of cancer death, accounting for 49.5% of all cancer deaths, were hematologic (14.2%), breast (13.6%), lung (13.2%), colorectal (8.5%), and liver (7.8%) cancer. The most common PoD was the hematology/oncology ward with 28.7% (n = 351), followed by the ICU with 27.8% (n = 340), the APCU with 23.8% (n = 291), and the HMC general medicine ward with 7.7% (n = 94). Individuals who died at home, in the ER, or in a long-term facility constituted only 0.4% (n = 5), 4.5% (n = 55), and 0.7% (n = 9) of the sample, respectively. Finally, those who died abroad and in private hospitals in Qatar accounted for 6.0% (n = 73) and 0.2% (n = 3) of the sample, respectively.

### Trend Analysis

**Figure 1:**
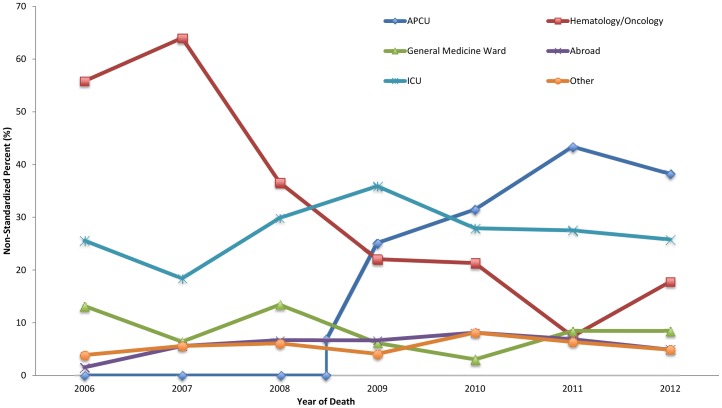
Place of cancer deaths in Qatar, 2006–2012, age-standardized against the UN mortality standard population [45].

Throughout the study period, the hematology/oncology ward was the most common PoD (32.4%; 95% CI 26.7–35.3), followed by the ICU (31.4%; 95% CI 28.7–34.3), APCU (26.9%; 95% CI 24.3–29.6) and general medicine ward (9.2%; 95% CI 7.6–11.1). Before 2009, the hematology/oncology ward was the most common PoD (56.4%; 95% CI 51.2–61.5), followed by the ICU (27.9%; 95% CI 23.4–32.8), general medicine ward (12.5%; 95% CI 9.3–16.3) and APCU (3.2%; 95% CI 1.7–5.5). After 2009, the APCU was the most common PoD (39.5%; 95% CI 35.9–43.2), followed by the ICU (33.3; 95% CI 29.8–36.9), hematology/oncology ward (19.7%; 95% CI 16.8–22.8) and general medicine ward (7.5%; 95% CI 5.7–9.7) (Fig. 1). Trend analysis for the APCU showed a constant increase (annual increase rate in the PoD of 0.057 per year; 95% CI 0.031–0.083; p<0.001), whereas the hematology/oncology ward showed a constant downward trend (annual decrease rate in PoD of −0.055 per year; 95% CI: −0.079–−0.031; p<0.001) (Table 2). No statistically significant changes occurred in the other PoDs.

**Table 2:** Chi-squared trend analysis for place of death.

Place of death	Rate of Change	95% CI	p-value
Hematology/oncology ward	−0.055	−0.079–−0.031	<0.001*
Acute palliative care unit	0.057	0.031–0.083	<0.001*
General medicine ward	0.0003	−0.021–0.022	0.974
Intensive care unit	0.006	−0.019–0.031	0.525
Abroad	0.008	−0.008–0.024	0.177
Other	−0.003	−0.019–0.013	0.590

*p<0.05.

### Factors associated with the place where cancer patients died

At the bivariate level, the risk of death at the APCU/hematology-oncology ward among females was 1.45 times higher than that among males (95% CI 1.13–1.85) (Table 3). Patients with hematological and liver cancer were less likely to die in the APCU/hematology-oncology ward (OR: 0.26, 95% CI 0.15–0.45 and OR: 0.48, 95% CI 0.27–0.88, respectively) than those with colorectal cancer. At the multivariate level, the same variables were significantly associated with death at the APCU/hematology-oncology ward, where females were more likely than males (OR: 1.37, 95% CI 1.03–1.82) and patients with hematological or liver cancer were less likely than those with colorectal cancer (OR: 0.28, 95% CI 0.16–0.48 and OR: 0.53, 95% CI 0.29–0.96, respectively).

**Table 3:** Unadjusted and adjusted odds ratios along with 95% CIs of variables associated with the place of death (APCU/hematology-oncology versus general medicine ward/ICU) in Qatar, 2006–2012.

	Unadjusted OR	95% CI	p-value	Adjusted OR	95% CI	p-value
**Age**			0.586			0.531
<65 years	1.00			1.00		
≥65 years	0.93	0.72–1.20		0.92	0.70–1.20	
**Sex**			0.003*			0.032*
Male	1.00			1.00		
Female	1.45	1.13–1.85		1.37	1.03–1.82	
**Nationality**			0.901			0.621
Qatari	1.00			1.00		
Non-Qatari	0.98	0.77–1.26		1.07	0.82–1.39	
**Cancer Diagnosis**						
Colorectal	1.00			1.00		
Hematological	0.26	0.15–0.46	<0.001*	0.28	0.16–0.48	<0.001*
Breast	1.00	0.58–1.72	0.987	0.85	0.48–1.50	0.577
Lung	1.01	0.58–1.74	0.985	1.13	0.64–1.98	0.682
Liver	0.48	0.27–0.88	0.018*	0.53	0.29–0.96	0.036*
Stomach	0.90	0.46–1.77	0.760	0.98	0.50–1.94	0.960
Pancreas	1.63	0.78–3.41	0.191	1.73	0.83–3.63	0.147
Other	0.80	0.50–1.30	0.372	0.84	0.52–1.36	0.475
**Year**			0.907			
<2009	1.00			1.00		
≥2009	0.98	0.76–1.27		0.94	0.72–1.23	0.662

*p<0.05.

Adjusted OR stratified by nationality. Qataris aged 65 years and older were less likely to die in the APCU/hematology-oncology ward (OR: 0.64, 95% CI 0.43–0.97) compared to those younger than 65 years (Table 4). Additionally, Qataris with hematologic and liver cancers were less likely to die in the APCU/hematology-oncology ward (OR: 0.18 p<0.001 and OR: 0.23, p = 0.002, respectively) than Qataris with colorectal cancer.

**Table 4:** Adjusted odds ratios and 95% CIs of variables associated with the place of death (APCU/hematology-oncology versus general medicine ward/ICU) in Qatar, 2006–2012, stratified by nationality.

	Qatari			Non Qatari		
	Adjusted OR	95% CI	p-value	Adjusted OR	95% CI	p-value
**Age**			0.036*			0.340
<65 years	1.00			1.00		
≥65 years	0.64	0.43–0.97		1.21	0.82–1.77	
**Sex**			0.441			<0.001*
Male	1.00			1.00		
Female	0.84	0.54–1.31		2.05	1.39–3.04	
**Cancer Diagnosis**						
Colorectal	1.00			1.00		
Hematological	0.18	0.07–0.47	<0.001*	0.41	0.20–0.83	0.013*
Breast	0.60	0.25–1.43	0.250	1.03	0.48–2.20	0.937
Lung	0.71	0.28–1.76	0.457	1.64	0.78–3.41	0.191
Liver	0.23	0.08–0.57	0.002*	1.09	0.49–2.46	0.831
Stomach	0.65	0.22–1.88	0.422	1.40	0.57–3.46	0.463
Pancreas	0.86	0.26–2.77	0.794	3.12	1.17–8.31	0.023*
Other	0.49	0.24–1.03	0.060	1.28	0.66–2.47	0.469
**Year**			0.344			0.516
**<**2009	1.00			1.00		
≥2009	0.82	0.54–1.24		1.12	0.79–1.60	

*p<0.05.

Among non-Qataris, females were more likely to die in the APCU/hematology-oncology ward than males (OR: 2.05 95% CI 1.39-3.04). Additionally, non-Qataris with hematological cancer were less likely to die in the APCU/hematology-oncology ward than those with colorectal cancer (OR: 0.41, 95% CI 0.20-0.83). However, non-Qataris with pancreatic cancer were more likely to die in the APCU/hematology-oncology ward than non-Qataris with colorectal cancer (OR: 3.12, 95% CI 1.17–8.31).

## Discussion

**Figure 2:**
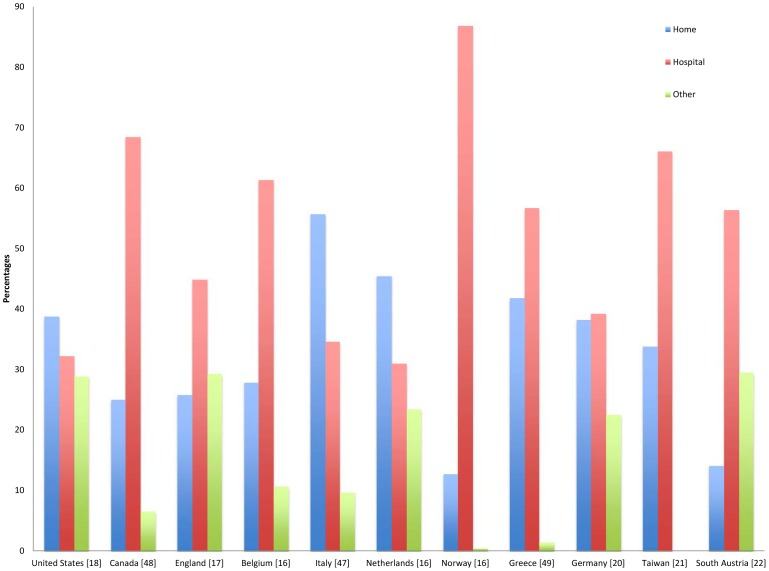
Worldwide comparison of places of cancer deaths.

Our population-based study found that an alarmingly high number of cancer deaths occurred in an acute care hospital setting, with only 0.4% of cancer deaths occurring at home. This is markedly different from the patterns of cancer deaths found in other developed countries (Fig. 2) [16]–[18], [20]–[22], [47]–[49]. Interestingly, this high rate of hospital deaths has been reported in one neighboring gulf country; a study in Kuwait analyzed the place of death of patients who died from cancer in 2009 and found that 98.7% of deaths occurred in hospital, whereas 1.0% occurred at home. To the best of our knowledge, this is the first population-based study in the Middle East analyzing the PoD of cancer patients over a several-year period.

After 2009, we observed a steady downward trend in deaths occurring in the hematology/oncology ward, mirrored by an upward trend in deaths occurring in the acute palliative care unit (APCU), which was confirmed by statistical modeling. This trend coincides with the launch of our APCU in July 2008, which provides terminal cancer patients with much-needed specialized palliative care toward the end of life. Before its initiation, both terminal patients and patients receiving active treatment were kept together in one ward, limiting the available resources and making healthcare delivery less than optimal. Despite this promising trend, there were no statistically significant changes in the other studied places of death, possibly because the palliative care service is still new and growing, and during the first three years, the admission criteria were constantly being modified. In that time, there was also a relative lack of awareness by physicians and patients regarding palliative care services in general. The percentage of home deaths remained very low and did not change throughout the study period.

A systematic review of 58 studies involving 1.5 million people from 13 countries revealed the association of 17 factors with the place of death. Six factors were found to be strongly associated with home deaths across 15 studies with high strength evidence, including patients' low functional status (OR range 2.29–11.1), patient preferences (2.19–8.38), use of home care (1.37–5.1) and its intensity/frequency (1.06–8.65), living with relatives (1.78–7.85), and extended family support (2.28–5.47). The authors further organized these factors into groups and listed the different variables in each group (reference); they found that environmental factors had the most influence on the place of death [50].

Perhaps the strongest factor influencing the remarkably low rate of home deaths in Qatar is the lack of alternative palliative care models, such as specialized home care and hospice services. The only palliative care service available to terminal cancer patients is the acute palliative care unit. As a result, patients have no choice other than the hospital setting to meet their end-of-life palliative care needs. Not surprisingly, the lack of alternative palliative care models has affected the dynamics of our APCU. A study conducted in 2012 found that the mean length of stay for patients admitted to the APCU was the highest reported in the world [46]. This issue makes the service provided in the APCU less than optimal because the actual resources available for acute cases are limited. However, the authors argued that our unit has more in common with chronic units than with an acute unit [46].

Several studies have documented the increased rate in home deaths (mirrored by a decreased rate of hospital deaths) in countries with established home care and hospice systems. In Edmonton, Canada, the establishment of alternative palliative care models decreased the percentage of cancer deaths in acute care hospitals from 63% to 32% (P<.001) and significantly decreased the average length of stay in both acute care facilities and the cancer center [51]. The additional new services provided that were responsible for the observed shift in the PoD included the following: establishment of hospice and home care services; palliative care teams, consisting of a consultant palliative care physician and nurse, who visit patients at home, hospice, community hospitals and continuing care facilities; increased funding for the delivery of 24-hour palliative care at home; and increased involvement of family physicians in delivering palliative care for patients at home and in hospice care. In a prospective, non-randomized study from Sweden, terminal cancer patients who received hospital-based advanced home care were significantly less likely to die in the hospital than those who received conventional hospital care (22% vs 63%, respectively; p<0.001) [52]. This report was further supported by a systematic review by Higgins et. al. where two of the six strongest factors associated with home death were use and intensity (the frequency of visits) of home care [50].

**Figure 3:**
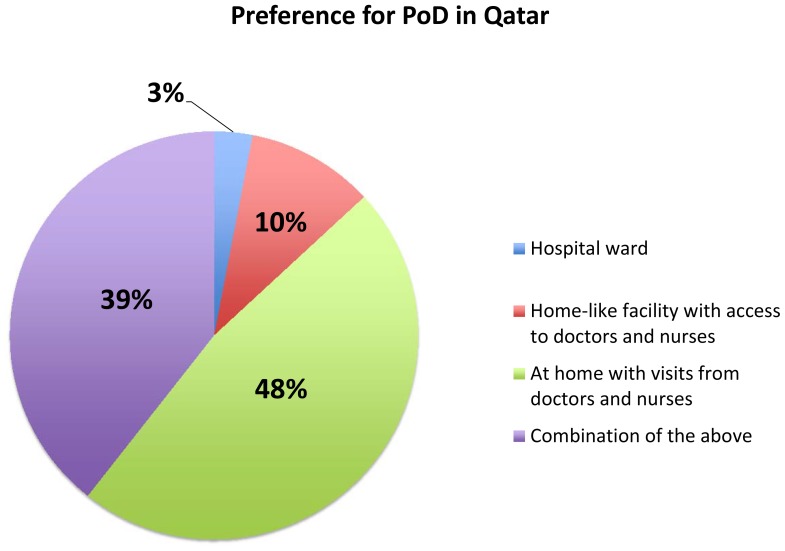
Survey of public sector workers conducted online from April–May 2011 regarding preference for end-of-life care and death. Reprinted from Qatar National Cancer Strategy [53] under a CC-BY license, with permission from Ara Darzi, original copyright 2011.

Given the lack of alternative palliative care modules for providing patients with the required palliative care, families tend to believe that the hospital setting, where trained physicians can provide adequate and specialized care, is the ideal place for their relatives in the end-of-life period. However, most patients would choose to have home care if they had sufficient resources at home for their palliative care needs. A public survey revealed that 47% of people wanted to spend their final days at home; 39% wanted access to a combination of home, hospital and home-like palliative care facilities; and only 3% wanted to spend their last days in a hospital ward (Fig. 3).

Our study revealed that older age (≥65 years) and hematologic and liver malignancies were associated with deaths in the ICU and general medicine ward in Qatari patients. In contrast, female sex and pancreatic cancers were associated with deaths in the APCU and hematology/oncology wards, whereas hematologic malignancy was associated with deaths in the ICU and general medicine wards.

There are two potential explanations for the increased risk of death in the general medicine ward and ICU in Qatari patients: one is cultural, and the other is associated with the location where patients receive their care. In Qatari culture, when the elderly become sick, they give permission for their immediate family members, such as the eldest sons, to perform the decision-making related to their care. As a result, the family's preference plays an important role in influencing the parents' place of death. The byproduct of this cultural practice is that the family often demands that everything be done for the parents. This becomes especially important when discussing DNR orders, which most Qatari families refuse. Consequently, elderly cancer patients are more aggressively managed until they suffer a major event, such as cardiac arrest, after which they are transferred to the ICU, where they subsequently die. A second potentially contributing factor is that elderly patients have multiple associated comorbidities, which are managed at Hamad General Hospital, where their condition may eventually deteriorate, leading to death. Furthermore, such elderly patients have a lower tolerance for cancer-related management, which puts them at an increased risk of complications in addition to their associated comorbidities.

Regarding the association between Qatari patients and liver cancer, it may be an interplay of factors related to the illness and the location where the patients receive care. Patients with liver cancer tend to have frequent exacerbations and admissions to the general medicine ward for medical and/or surgical management, such as relief of biliary obstruction, peritoneal tapping, and treatment for peritonitis. Eventually, while they are being managed, they tend to decompensate and pass away at that location. Another possible reason may be the availability of chemoembolization, which is performed by the interventional radiologists who are situated at Hamad General Hospital. Some patients may develop complications from the procedure, especially those who have poor performance status, and then rapidly decompensate and pass away. Finally, another possible reason is that many patients present very late in the course of the disease; they are admitted to the general medicine ward and may deteriorate rapidly and pass away during the investigation of their presenting illness. Nevertheless, further research is required to elucidate whether other factors are involved.

The association between non-Qatari patients and pancreatic cancer may be related to the poor prognosis associated with the disease. Most patients have either locally advanced or metastatic cancer at initial diagnosis, and the prognosis is poor, even in those with potentially resectable disease. Despite the use of potentially curative resection, the five-year survival following surgery is only approximately 20 percent [54]–[57]. As a result, oncologists at our center tend to discuss the end of life with patients early, resulting in more referrals to our APCU.

Our study also found that hematologic malignancy was associated with deaths at the ICU and general medicine wards among both Qatari and non-Qatari patients. The reasons for the high hospital death rate of patients with hematologic malignancies are complex and still under investigation, but may be related to the multiple treatment options available, even at advanced stages, to patients with hematologic malignancies. As a result, the referral/transition to palliative care is often not so apparent [58]. A recent study found that the time from diagnosis to death was a major factor influencing the place of death, with patients who died within three months of diagnosis dying in the hospital [59]. This pattern was associated with the disease sub-type, with more aggressive malignancies associated with earlier deaths, likely as a result of rapid progression without sufficient time to transition the patient to palliative care or as a result of complications of treatment [59]. A systematic review of factors influencing death at home among cancer patients also found that hematologic malignancies were associated with a lower risk of dying at home (OR 0.34–0.61) [50].

### Strengths and Limitations

The strengths of this study lie in its population-based design. The data collected are inclusive of all individuals who died from cancer as the underlying cause of death during the study period. Therefore, the findings can be directly applied to inform national policies and allocate additional resources to end-of-life care.

There are several limitations to our study. First, our odds ratio analysis groups were not ideal; most place of death studies evaluate associated factors between home/hospice and hospital deaths. Second, we did not have data on individual patients' preferences for the place of death or a clinical indication of the most appropriate place of death. Nevertheless, at the population level, the hospital is consistently considered the least preferred place of death, independent of many factors such as diagnosis, country, and setting [33]. Finally, we were not able to provide a potential explanation for the association between female non-Qataris and death at NCCCR or why liver and pancreatic malignancies were associated with nationality. Further research is needed to illuminate these findings.

## Conclusion

In conclusion, a high percentage of cancer deaths in Qatar occur in the hospital. Because home is the preferred place of death for most people, effective home care and hospice programs are required to enable patients to remain at home in accordance with their preference. Furthermore, caring for such patients in the hospital will become unsustainable in terms of the capacity, cost, and patient/caregiver satisfaction/quality of life.
